# Glucose and insulin dynamics following low-carbohydrate and low-fat diets in females with lipedema and obesity

**DOI:** 10.3389/fcell.2026.1893868

**Published:** 2026-09-15

**Authors:** Julianne Lundanes, Anne Sofie Fischer, Simon Nitter Dankel, Jens Juul Holst, Catia Martins, Siren Nymo

**Affiliations:** 1 Nord-Trøndelag Hospital Trust, Clinic of Surgery, Namsos Hospital, Namsos, Norway; 2 Department of Clinical and Molecular Medicine, Faculty of Medicine and Health Sciences, Norwegian University of Science and Technology (NTNU), Trondheim, Norway; 3 Department of Clinical Medicine, Centre for Nutrition, University of Bergen, Bergen, Norway; 4 Department of Clinical Science, University of Bergen, Bergen, Norway; 5 NNF Center for Basic Metabolic Research and Department of Biomedical Sciences, The Panum Institute, University of Copenhagen, Copenhagen, Denmark; 6 Department of Nutrition Sciences, University of Alabama at Birmingham (UAB), Birmingham, AL, United States; 7 Department of Public Health and Nursing, Faculty of Medicine and Health Sciences, Norwegian University of Science and Technology (NTNU), Trondheim, Norway; 8 Regional Center of obesity Research and Innovation, Clinic of Surgery, St. Olavs Hospital, Trondheim, Norway

**Keywords:** C-peptide, GIP, insulin resistance, insulin sensitivity, ketosis

## Abstract

**Introduction:**

Low-carbohydrate diets (LCDs) have been suggested as a potential treatment option for females with lipedema, due to their superiority in reducing pain, possibly through improvements in insulin dynamics.

**Objectives:**

The objective of this secondary analysis was to compare changes in markers of glucose and insulin dynamics after a low-carbohydrate versus low-fat low-energy diets in females with lipedema and obesity. A secondary objective was to investigate potential associations between changes in glucose and insulin dynamics and changes in pain.

**Methods:**

Females with obesity and lipedema were randomized to either an LCD or a low-fat low-energy diets (1200 kcal/day for both; 75 vs. 180 g carbohydrates/day) for 8 weeks. Basal and postprandial (total and incremental area under the curve (tAUC and iAUC)) concentrations of glucose, insulin, glucose-dependent insulinotropic polypeptide (GIP), and C-peptide were measured before and after the intervention, and HOMA-IR, Matsuda index and insulin clearance calculated.

**Results:**

A total of 70 females (35 in each group, age 47 ± 11 years, BMI 37 ± 5 kg/m^2^) were included in the analysis. Both groups lost body weight, but with a significantly greater weight loss in the LCD group. Both groups showed ed a reduction in basal glucose, C-peptide, and insulin concentrations, with a greater reduction in glucose on the LCD. A significant increase in glucose iAUC, C-peptide iAUC, insulin clearance, and Matsuda index was observed in the LCD group only. Both groups experienced an increase in GIP tAUC, as well as a reduction in HOMA-IR, with no significant differences between groups. No associations were seen between changes in glucose and insulin measures and changes in pain.

**Conclusion:**

Both diets improved insulin dynamics in females with lipedema and obesity, despite additional benefits in the LCD group. However, changes in the measured indices of insulin dynamics were not associated with pain reduction, suggesting that the effects of the LCD on pain and systemic metabolic regulation may involve other pathways.

## 1 Introduction

Lipedema is a chronic disease in females characterized by a symmetrical disproportional distribution of subcutaneous adipose tissue, and pain and feelings of heaviness in the extremities (Faerber et al., 2024). Females with lipedema report lower quality of life (QoL) compared to the general population (Falck et al., 2022; Romeijn et al., 2018). The prevalence of lipedema is unknown, the etiology and pathophysiology remain poorly described, and patients lacked access to standardized care within the public healthcare system (Ekerhovd et al., 2025). In terms of adipose tissue function, lipedema is characterized by immune cell infiltration, interstitial fibrosis, and vascular and lymphatic abnormalities (Kruppa et al., 2023; Streubel et al., 2024; Poojari et al., 2022). The combined effects of these abnormalities may influence pain through mechanisms such as increased interstitial pressure, altered tissue mechanics, and sensitization of nociceptive pathways, at least in part suggested to involve neuronal hypersensitivity in adjacent skin (Chakraborty et al., 2022). Because lipedema affects females, mechanisms of altered signaling via estrogens and other sex hormones have been proposed (Lüchinger et al., 2026), but the potential roles of other endocrine signals in lipedema are largely unknown.

We previously showed that ketogenic low-carbohydrate diets (LCDs) can relieve pain in females with lipedema (Sørlie et al., 2022; Lundanes et al., 2024a). Several mechanisms have been proposed to explain symptom improvement following carbohydrate restriction, including alterations in fatty acid metabolism, ketone body signaling, inflammation, vascular and lymphatic function, adipose tissue remodeling, and improvements in insulin sensitivity (Verde et al., 2023; Sanlier and Baltacı, 2025; Keith et al., 2021; Atabilen et al., 2026). Among these potential mechanisms, improved glucose-insulin regulation may contribute to symptom relief, as insulin resistance has been implicated in metabolic dysfunction and inflammation (Keith et al., 2021). In overweight and obesity, LCDs consisting of 50-130 g of carbohydrates per day (or 10-26 E%) have been shown to significantly reduce basal glucose and insulin concentrations, and insulin resistance (Feng et al., 2025). However, while the impact of weight loss in general on insulin dynamics in females with lipedema has been investigated previously (Cifarelli et al., 2025), few studies have looked at LCDs in particular, and those studies suffer from methodological constrains, namely, the lack of a high-carbohydrate low-fat diet control group (Jeziorek et al., 2023) and a small sample size (Sørlie et al., 2022). Further studies are needed to clarify the potential role of macronutrient composition of the diet, in particular LCDs versus low-fat diets, on insulin dynamics in females with lipedema and obesity and to investigate the potential role of insulin dynamics on improvements in pain perception with LCDs in this patient group.

Therefore, the primary objective of this study was to compare changes in glucose and insulin dynamics, including basal and postprandial concentrations of glucose, insulin, glucose-dependent insulinotropic polypeptide (GIP), and C-peptide, as well as indexes of insulin sensitivity, such as the HOMA-IR, and the Matsuda index, and insulin clearance, after a low-carbohydrate versus low-fat low-energy diet in females with lipedema and obesity. Secondary objectives were to investigate potential associations between changes in glucose and insulin dynamics and changes in pain.

## 2 Methods

### 2.1 Study design

This manuscript describes secondary analyses of a randomized controlled trial (RCT) comparing the effects of an LCD versus a low-fat low-energy diet on pain and QoL in females with lipedema (Lundanes et al., 2024a). The study was approved by the Regional Ethical Committee of Central Norway (REK 93888) and registered in Clinicaltrials.gov (NCT04632810). All participants provided written informed consent in line with the Helsinki Declaration, before entering the study. Participants were randomized (1:1) by block randomization with stratification by body mass index (BMI) categories (30.0-34.9, 35.0-39.9, 40.0-44.9 kg/m^2^). Randomization was performed by a web-based randomization system developed and administered by the faculty of Medicine and Health Sciences, Norwegian University of Science and Technology, Trondheim, Norway. Data collection was done using eFORSK, a web-based system developed and administered by Helse Midt-Norge IT (Central Norway Regional Health Authority’s IT department).

### 2.2 Study population

Females diagnosed with lipedema and obesity (BMI between 30 and 45 kg/m2), aged 18 - 75 years old, and weight stable for the last 3 months (±3 kg) were invited to participate in the study. Participants were diagnosed with lipedema by physiotherapists before inclusion, and type and stage of lipedema were assessed at baseline by study personnel. Exclusion criteria included acute and chronic kidney disease/failure, previous bariatric surgery, malignant disease, infectious disease, diabetes, psychosocial disorders, breastfeeding, pregnancy, current medication known to affect body weight and/or appetite, not mastering a Scandinavian language, and enrollment in another obesity/lipedema treatment program (except from regular physiotherapy, including compression garments/pulsator, etc.).

### 2.3 Dietary intervention

The intervention was run at the Obesity out-patient clinic at St. Olavs’ University Hospital (Trondheim, Norway) from June 2021 to May 2023. The diets were food-based and were matched for energy (1200 kcal/day) and protein (60 g, energy percentage (E%) 20 E%) but differed in carbohydrate and fat content. The LCD consisted of 75 g of carbohydrates (25 E%) and 73 g of fat (55 E%), while the low-fat diet consisted of 180 g of carbohydrates (60 E%) and 27 g of fat (20 E%). The LCD consisted mainly of meat/poultry/fish, eggs, nuts, small amounts of low-carbohydrate fruits and vegetables, and dairy. Butter and whole-fat dairy were replaced with healthy fats such as olive oil and food rich in polyunsaturated fatty acids (PUFAs). The control diet consisted mainly of whole grains, including bread, pasta, and rice; legumes, fruits, and vegetables; and moderate amounts of eggs, meat/poultry/fish, and low-fat dairy (Lundanes et al., 2024a). Participants had to follow the diets for 8 weeks. The dietary plans were individually adjusted by registered dietitians to account for food preferences, intolerances, and allergies. Participants were advised to take a multivitamin-mineral supplement because the prescribed energy intake of 1200 kcal/day (approximately 5.0 MJ/day) was below the threshold of 6.5 MJ/day defined as a low energy intake in the Norwegian dietary reference values (Helsedirektoratet. Anbefalinger om kosthold, 2014). They were also recommended to drink a minimum of 2 L of calorie-free drinks daily (limiting artificially sweetened beverages).

#### 2.3.1 Compliance

Participants had weekly follow-ups, during which body weight was measured, ketosis assessed (see below) and potential side effects of the diets discussed, aiming at enhancing compliance and preventing dropouts. Necessary changes and adjustments in the diets were made, within the constraints of energy and macronutrient distribution. Participants were asked to fill out daily food records throughout the whole intervention period, where they reported which pre-specified meal they ate, and if there were any deviations. These were then analyzed for intake of energy (kcal/day) and macronutrients (g/day, E %), using a web-based analysis program (Kostholdsplanleggeren. Norwegian Directorate of Health, 2022) based on the Norwegian Food Composition Table (Norwegian Food Safety Authority and The Norwegian Directorate of Health, 2019) and discussed at the weekly follow-ups.

Ketostix® reagent test strips (Ascensia Diabetes Care Holdings AG, Basel, Switzerland) were used in the weekly follow-ups to measure urinary acetoacetate (AcAc) concentration. A cutoff level <0.5 mmol/L was used for negative AcAc. ß-hydroxybutyrate (ßHB) concentration was measured in capillary blood using finger-pricks (Freestyle Precision Neo, Abbott, CA, United States) and Freestyle β-ketone reagent strips (Abbott, CA, United States) at baseline, week 5 and week 9. Participants with a ßHB plasma concentration ≥0.3 mmol/L were defined as being in nutritional-induced ketosis.

The participants were asked not to change their physical activity levels throughout the study period, and physical activity was objectively assessed at baseline and week 9, as previously described (Lundanes et al., 2024b).

### 2.4 Outcome variables

The following variables were collected at baseline and post-intervention (week 9).

#### 2.4.1 Anthropometrics

Circumferences of the waist, hip, thigh, and calf were measured to the nearest 0.1 cm using a metric tape. Participants wore only underwear during the assessments. Each measurement was performed three times, and the average value was used in the analysis. Waist circumference was measured at the midpoint between the lowest rib and the upper border of the iliac crest. Thigh circumference was measured at the midpoint between the knee and the hip, while hip and calf circumferences were measured at its widest point. The height at which the thigh, calf, and hip measurements were taken was recorded to ensure consistent placement at baseline and at week 9. Measurements were taken by the same study personnel at both baseline and week 9 to reduce inter-observer variability. Waist to height ratio (WHtR) was calculated as waist circumference (cm) divided by height (cm) (Brenner et al., 2023). Data on body composition has already been published. Body composition was assessed using bioelectrical impedance analysis (InBody 720, Seoul, South Korea) at baseline and week 9 in all participants (Lundanes et al., 2024a), while regional calf subcutaneous adipose tissue was assessed using MRI in a subset of participants (Lundanes et al., 2024c).

#### 2.4.2 Laboratorial analyses

Blood samples were collected before and after a standardized breakfast (600 kcal) (30, 60, 90, 120 and 150 min postprandially, and additionally at 15 and 45 min for the measurements of glucose and C-peptide), as previously described (Lundanes et al., 2024b). Glucose and C-peptide were measured from heparin-gel tubes and analyzed using standardized methods at St. Olavs hospital. Insulin and GIP were measured from EDTA-tubes (10 ul DPPIV (Merck Millipore, Germany) and 10 ul pefabloc (Roche Diagnostic, Germany) were added to 1 ml of whole blood for the measurement of insulin and 11 ug aprotinin was added to 4 ml of whole blood for GIP analysis). The samples were then centrifuged at 18° C, for 10 min at 3200 RPM. Plasma was retrieved and then stored at−80° C until further analysis. Insulin was measured with a Human Metabolic Hormone Magnetic Bead Panel (LINCOplex Kit, Millipore, St. Louis, MO, United States) and GIP with an in house RIA (Deacon and Holst, 2009) at the University of Copenhagen, Denmark. Intra- and inter-assay coefficient of variation (%CV) were < 13 and < 20 for insulin.

##### 2.4.2.1 Measures of insulin resistance

Insulin resistance was estimated using the Homeostatic Model Assessment for Insulin Resistance (HOMA-IR) and the Matsuda index. HOMA-IR was calculated as fasting glucose (mmol/L) × fasting insulin (µIU/mL)/22.5 (Levy et al., 1998). Insulin was converted from pg/mL to µIU/mL by multiplying with 0.0287. To determine Matsuda Index, the formula 10000/(√basal insulin (µU/mL) * basal glucose (mg/dL) * (mean postprandial insulin (µU/mL) * mean postprandial glucose (mg/dL) was used (Matsuda and DeFronzo, 1999). Glucose was converted from mmol/L to mL/dL by multiplying by 18. Hepatic insulin clearance was estimated as the molar ratio of C-peptide to insulin concentrations in postprandial (tAUC) states.

#### 2.4.3 Pain

Changes in pain were assessed with the Brief pain inventory, as previously described (Lundanes et al., 2024a).

### 2.5 Statistical analysis

Statistical analysis was performed using Stata 19 (StataCorp LLC, Texas, United States), data presented as means and standard deviations (SD), and results from statistical tests as estimated marginal means (EMM) with corresponding 95% confidence intervals (CI) and p values. Statistical significance was set at P < 0.05. Total and incremental area under the curve (tAUC and iAUC) were calculated from 0 to 150 min using the trapezoid rule. Group differences in the changes from baseline in all outcome variables (fasting values, tAUC and iAUC) were estimated by linear mixed-effect models. The fixed part was specified in terms of two dummy variables: one for time (within low-fat diet group) and one for group differences (LCD vs. low-fat diet) post intervention (week 9), since baseline means can be assumed to be the same given the randomized design of the study (Coffman et al., 2016; Twisk et al., 2018). The mean difference in changes from baseline is equivalent to the estimated mean group difference post intervention (between groups). Linear combination of the parameters in the models was assessed to estimate changes in the LCD group. A random intercept for participants was included to account for within-participant correlations. To adjust for weight loss, multiple linear regression was performed with changes in glucose and insulin dynamic markers as dependent variable (week 9 minus baseline), and weight loss and group as independent variables. Residuals were checked for normality using histograms and Shapiro wilk-test. The association between changes in the different markers of insulin dynamics and reductions in pain were determined with Pearson or Spearman correlation between change in pain (week 9 minus baseline) and changes in glucose and insulin dynamic variables (week 9 minus baseline), Figures were generated using GraphPad Prism (Version 10.0.2 for Windows, GraphPad Software, Boston, Massachusetts, United States).

## 3 Results

All participants included in the primary analysis were also included in this secondary analysis. Seventy females with lipedema and obesity with a mean age of 47 ± 11 years, and mean BMI of 37 ± 5 kg/m2 were included, 35 in each group. Baseline demographic and anthropometric characteristics were previously published (Lundanes et al., 2024a).

### 3.1 Dietary adherence and ketones

Dietary adherence data have already been published, but briefly the LCD group had a significantly lower carbohydrate and fiber intake and higher fat intake, as in line with the study design. However, the LCD group had a significantly higher protein intake compared to the low-fat diet group. ßHB concentrations were 0.6 ± 0.4 mmol/L in the LCD group and 0.2 ± 0.1 mmol/L in the low-fat diet group at week 5 and 0.7 + 0.5 mmol/L in the LCD group and 0.2 + 0.1 mmol/L in the low fat diet group at week 9 (Lundanes et al., 2024a).

### 3.2 Anthropometrics

Both the LCD and low-fat diet groups lost weight, but with a significantly greater weight loss for the LCD group (average −10 kg and −7 kg, respectively, P < 0.001) (Lundanes et al., 2024a). Changes in anthropometric measurements are presented in Table 1. Both groups had a significant reduction in waist, hip and thigh circumference, as well as WHtR, while only the LCD group had a reduction in calf circumference. The reduction in hip and calf circumference was significantly greater in the LCD group compared to the low-fat diet group. As previously published, the LCD group experienced a greater reduction in pain compared with the low-fat diet group (Lundanes et al., 2024a).

**TABLE 1:** Anthropometrics in both group at baseline and week 9, changes within group and differences between groups.

​		BL	W9	Difference from BL to w9			Difference in change between groups		
		Mean (SD)	Mean (SD)	EMM	95% CI	P value	EMM	95% CI	P value
Waist circumference, cm	LCD	102.78 (15.41)	93.90 (10.86)	−9.08	−12.49 to −5.67	**<0.001**	−2.95	−7.70 to 1.81	0.224
	Low-fat diet	103.12 (14.10)	96.08 (14.77)	−6.13	−9.65 to −2.61	**0.001**			
Hip circumference, cm	LCD	123.50 (10.34)	116.04 (8.63)	−7.48	−9.43 to −5.53	**<0.001**	−3.30	−6.07 to −0.54	**0.019**
	Low-fat diet	122.31 (15.23)	119.47 (12.19)	−4.18	−6.19 to −2.17	**<0.001**			
Thigh circumference, cm	LCD	69.25 (7.98)	64.49 (7.31)	−4.69	−5.95 to −3.42	**<0.001**	−1.73	−3.52 to 0.06	0.058
	Low-fat diet	69.31 (7.16)	66.01 (6.15)	−2.95	−4.26 to −1.65	**<0.001**			
Calf circumference, cm	LCD	46.62 (4.90)	44.46 (4.79)	−2.18	−3.74 to −0.61	**0.006**	−2.40	−4.52 to 0.27	**0.027**
	Low-fat diet	46.91 (5.45)	46.90 (6.46)	0.22	−1.39 to 1.83	0.789			
WHtR	LCD	0.62 (0.10)	0.56 (0.06)	−0.05	−0.08 to −0.03	**<0.001**	−0.02	−0.05 to 0.01	0.202
	Low-fat diet	0.62 (0.09)	0.58 (0.09)	−0.04	−0.06 to −0.01	**0.001**			

Data presented as mean (SD) and results from linear mixed models are presented as estimated marginal means (EMM), 95% confidence interval and p value. N = 35 in low-fat diet group at baseline (BL) and n = 28 at week 9 (w9). N = 33 in LCD, group at BL, and n = 32 at week 9. Significant p values are bolded. CI: Confidence interval. LCD: low carbohydrate diet. WHtR: waist to height ratio.

### 3.3 Glucose, insulin and incretines

Changes in several markers of insulin dynamics in both diet groups are presented in Table 2. Basal glucose concentrations decreased in both diet groups by −0.5 mmol/L in LCD group (P < 0.001) and −0.2 mmol/L in low-fat diet group (P = 0.022), with a significantly greater reduction in the LCD group compared to the low-fat diet group (P = 0.031). An increase in glucose iAUC was seen only in the LCD group (103.1 mmol/L*min, P < 0.001), which was significantly different from the low-fat diet group (P < 0.001). Both LCD and low-fat diet groups showed reductions in basal C-peptide (−0.2 nmol/L and −0.1 nmol/L, respectively) and insulin concentrations (−5.7 µIU/mL and −4.6 µIU/mL, respectively). An increase in C-peptide iAUC was only seen in the LCD group (37.5 nmol/L*min, P = 0.011), and this increase was significantly greater compared to that seen in low-fat diet group (P = 0.018). In contrast, a reduction in insulin tAUC was observed only in the low-fat diet group (−2127.6 µIU/mL *min, P = 0.004), but with no statistically significant differences between diet groups. No changes were seen for basal GIP concentrations; however, both diet groups experienced an increase in GIP tAUC, with no statistically significant differences between groups.

**TABLE 2:** Basal and postprandial plasma concentrations of glucose, insulin, c-peptide and GIP in both diet groups over time.

​		Baseline	Week 9	Difference from BL to w9			Difference between groups in changes over time		
		Mean (SD)	Mean (SD)	EMM	95% CI	P value	EMM	95% CI	P value
Glucose									
Fasting, mmol/L	LCD	5.4 (0.7)	5.0 (0.5)	−0.5	−0.6 to −0.3	**<0.001**	−0.3	−0.5 to −0.0	**0.031**
	Low-fat diet	5.3 (0.6)	5.1 (0.5)	−0.2	−0.4 to −0.0	**0.022**			
tAUC, mmol/L*min	LCD	843.8 (120.4)	873.3 (112.9)	34.5	−2.5 to 71.4	0.067	64.9	14.2 to 115.7	**0.012**
	Low-fat diet	819.8 (148.4)	790.4 (122.0)	−30.5	−69.3 to 8.3	0.124			
iAUC, mmol/L*min	LCD	28.0 (76.8)	129.9 (107.9)	103.1	69.7 to 136.4	**<0.001**	103.9	59.5 to 148.4	**<0.001**
	Low-fat diet	33.5 (95.2)	26.5 (103.5)	−0.9	−36.1 to 34.3	0.961			
C-peptide									
Fasting, nmol/L	LCD	0.9 (0.4)	0.7 (0.4)	−0.2	−0.3 to −0.1	**<0.001**	−0.1	−0.2 to 0.0	0.232
	Low-fat diet	0.9 (0.4)	0.8 (0.4)	−0.1	−0.2 to −0.0	**0.007**			
tAUC, nmol/L*min	LCD	344.8 (112.1)	352.3 (106.0)	9.8	−25.3 to 45.0	0.584	35.6	−13.1 to 84.4	0.152
	Low-fat diet	328.0 (143.8)	305.5 (132.2)	−25.8	−63.0 to 11.5	0.175			
iAUC, nmol/L*min	LCD	208.9 (68.9)	243.1 (86.5)	37.9	8.6 to 67.2	**0.011**	47.5	8.3 to 86.8	**0.018**
	Low-fat diet	196.6 (87.4)	191.7 (98.5)	−9.6	−40.7 to 21.5	0.545			
Insulin									
Fasting, µIU/mL	LCD	22.5 (11.6)	17.9 (9.3)	−5.7	−8.6 to −2.7	**<0.001**	−1.0	−5.2 to 3.1	0.623
	Low-fat diet	23.1 (16.6)	19.4 (12.8)	−4.6	−7.7 to −1.5	**0.004**			
tAUC, µIU/mL*min	LCD	10,501.1 (6050.5)	7103.4 (5069.2)	−1121.7	−473.2 to 229.7	0.104	1005.8	−902.9 to 2,914.6	0.302
	Low-fat diet	9632.6 (6848.8)	6087.4 (4851.8)	−2,127.6	−560.4 to −697.4	**0.004**			
iAUC, µIU/mL*min	LCD	9698.2 (4877.0)	7016.8 (4238.1)	−173.7	−1379.4 to 1032.0	0.778	1266.5	−420.3 to 2,953.3	0.141
	Low-fat diet	7813.8 (5117.4)	4955.0 (3995.9)	−1440.2	−719.5 to −160.9	**0.027**			
GIP									
Fasting, pmol/L	LCD	12.2 (5.3)	12.6 (16.3)	1.2	−3.3 to 5.7	0.602	0.8	−4.9 to 6.4	0.791
	Low-fat diet	10.5 (6.5)	11.6 (14.0)	0.4	−4.3 to 5.1	0.858			
tAUC, pmol/L*min	LCD	8914.7 (919.7)	10,228.5 (3481.0)	1600.6	802.3 to 398.9	**<0.001**	642.2	−470.1 to 1754.5	0.258
	Low-fat diet	7669.1 (510.1)	8659.2 (3150.4)	958.4	125.0 to 1791.8	**0.024**			
iAUC, pmol/L*min	LCD	7067.3 (854.4)	8319.6 (3859.3)	1552.8	532.2 to 573.4	**0.003**	896.8	−482.6 to 2,276.1	0.203
	Low-fat diet	6123.6 (542.2)	6945.4 (2,2945.9)	656.0	−413.2 to 1725.3	0.229			
Matsuda index									
​	LCD	3.9 (3.3)	4.3 (2.7)	0.9	0.4 to 1.4	**0.001**	0.4	−0.4 to 1.1	0.304
	Low-fat diet	4.2 (3.0)	4.8 (2.5)	0.5	−0.0 to 1.1	0.063			
HOMA-IR									
​	LCD	5.4 (3.1)	4.0 (2.3)	−1.7	−2.4 to −0.9	**<0.001**	−0.4	−1.5 to 0.7	0.465
	Low-fat diet	5.6 (4.0)	4.4 (3.1)	−1.3	−2.1 to −0.4	**0.003**			
Insulin clearance									
​	LCD	8.2 (9.2)	7.5 (4.9)	0.8	0.0 to 1.5	**0.046**	0.2	−0.9 to 1.3	0.067
	Low-fat diet	6.5 (2.5)	7.3 (2.5)	0.5	−0.3 to 1.3	0.193			

Data presented as mean (SD). n = 32 in LCD, group at baseline and n = 32 at week 9, and n = 34 in low-fat diet group at baseline and n = 28 at week 9. Significant p values are bolded. BL: Baseline. W9: Week 9. EMM: estimated marginal means; CI: confidence interval; tAUC: total area under the curve; iAUC: incremental area under the curve; LCD: low-carbohydrate diet; GIP: glucose-dependent insulinotropic polypeptide. HOMA-IR: homeostasis model assessment of insulin resistance.

### 3.4 Insulin sensitivity and clearance

Both the LCD and low-fat diet groups showed a reduction in HOMA-IR (−1.7, P < 0.001 and −1.3, P = 0.003, respectively), while only the LCD group had an improvement in Matsuda index (0.9, P = 0.001). However, no statistically significant differences between groups were seen for HOMA-IR or Matsuda index. An increase in postprandial insulin clearance was found in the LCD group only (0.8, P = 0.046), but with no statistically significant between-group differences.

Changes in glucose and insulin dynamic variables after adjusting for weight loss are presented in Supplementary Table S1. These analyses did not change the overall results, except that the difference between groups for glucose tAUC and C-peptide tAUC was no longer statistically significant.

### 3.5 Associations with pain

No associations were found between changes in any marker of glucose and insulin dynamics and changes in pain (see Table 3).

**TABLE 3:** Correlations between changes in pain now and changes in glucose and insulin dynamic variables.

​	Change in pain			
	LCD		Low-fat diet group	
	Correlation coefficient	P value	Correlation coefficient	P value
Glucose				
Fasting, mmol/L	0.267	0.154	−0.189	0.336
tAUC, mmol/L*min	0.121	0.533	−0.113	0.575
iAUC, mmol/L*min	−0.060	0.757	0.040	0.841
C-peptide				
Fasting, nmol/L	0.222	0.230	−0.129	0.514
tAUC, nmol/L*min	0.049^1^	0.807	0.157^1^	0.441
iAUC, nmol/L*min	0.0611^1^	0.761	−0.079^1^	0.699
Insulin				
Fasting, µIU/mL	0.220^1^	0.268	−0.059^1^	0.775
tAUC, µIU/mL*min	0.046	0.815	−0.342	0.087
iAUC, µIU/mL*min	−0.017	0.931	−0.343	0.086
GIP				
Fasting, pmol/L	0.270^1^	0.172	−0.171^1^	0.775
tAUC, pmol/L*min	0.109^1^	0.586	−0.098^1^	0.633
iAUC, pmol/L*min	0.093^1^	0.642	0.203^1^	0.319
Matsuda index				
−0.153		0.437	−0.070	0.735
HOMA-IR				
0.235		0.219	−0.312	0.121
Insulin clearance				
0.066^1^		0.742	−0.065^1^	0.752

Data analyzed using Pearson or

1 spearman correlation depending on normality. CI: Confidence interval. GIP: glucose-dependent insulinotropic polypeptide. HOMA-IR: homeostasis model assessment of insulin resistance.

## 4 Discussion

The primary objective of this study was to compare changes in glucose and insulin dynamics after a low-carbohydrate versus a low-fat low-energy diet in females with lipedema and obesity. Secondary objectives were to investigate potential associations between changes in glucose and insulin dynamics and changes in pain. In a randomized controlled trial of females with lipedema and obesity, we here found that both LCD and low-fat low-energy diets lowered basal glucose, insulin, and C-peptide concentrations, but with a significantly greater reduction in basal glucose following the LCD. Moreover, only the LCD group showed an increase in postprandial glucose and C-peptide concentrations, Matsuda index, and insulin clearance, although only postprandial glucose and C-peptide concentrations differed significantly between the two diets. However, the difference between groups in postprandial C-peptide was no longer significant after adjusting for weight loss. Postprandial insulin concentrations were significantly reduced only after the low-fat diet, but with no between-group differences. HOMA-IR decreased and postprandial GIP concentrations increased in both groups with no differences between groups. No significant associations were found between changes in insulin dynamics and changes in pain.

In the LCD group, postprandial C-peptide concentrations increased, while postprandial insulin concentrations remained unaltered, indicating increased hepatic insulin clearance since C-peptide has a half-life of around 30 min compared to 5 min for insulin. This occurred together with improved insulin sensitivity, as assessed by the Matsuda index in the LCD group. These data indicate that less insulin was needed after the LCD to dispose the ingested meal. Thus, while we did not measure tissue-specific insulin sensitivity, our data suggest that site-specific reduction of adipose tissue in lipedema on the LCD, as reflected ed by reduced hip- and thigh circumference, occurred alongside improvements in whole-body insulin sensitivity. However, whether these changes reflect improved adipose tissue insulin sensitivity or adipose tissue function remains speculative and should be confirmed using direct physiological and molecular assessments of adipose tissue in future studies.

To our knowledge, our study is the first to report a diet-specific improvement in insulin clearance in females with lipedema. Insulin clearance is a feature of metabolic dysregulation as observed in type 2 diabetes (Zaharia et al., 2023), Studies suggest that females with lipedema generally exhibit better insulin sensitivity compared with BMI-matched controls (Faerber, 2018; Jeziorek et al., 2025; Nankam et al., 2022), and that the adipose tissue accumulation in lipedema is a healthier adipose tissue expansion compared with visceral adiposity (Nankam et al., 2022; Mühlberg et al., 2026). At the same time, lipedema tissue has been hypothesized to display local insulin resistance, despite whole body insulin sensitivity (Keith et al., 2021), but neither adipose tissue insulin sensitivity nor tissue-specific insulin signaling were assessed in the present study. Notably, the participants in the present study presented with insulin resistance at baseline, commonly defined as HOMA-IR > 2.5 (Jeziorek et al., 2025; Parcha et al., 2022). Mean HOMA-IR values were 5.4 and 5.6, in the LCD and low-fat diet groups, respectively. This metabolic profile likely reflects the higher BMI (37 kg/m^2^ versus 31 kg/m^2^) and waist circumference (103 cm versus 97 cm) of the participants in the present study compared with those in the study by Jeziorek et al., who reported normal HOMA-IR values (Jeziorek et al., 2023). Similarly, Faerber et al., reported HOMA-IR within the normal range (2.7), in a cohort with a lower baseline body weight (Faerber, 2018). The higher HOMA-IR at baseline in the present study may have given a greater potential for improvement in insulin sensitivity, which should be kept in mind in comparison with other studies.

Lowered HOMA-IR was seen on both diets, but the increased Matsuda index and insulin clearance were specifically seen on the LCD, which might partly be ascribed to the significantly greater weight loss in the LCD group. Cifarelli et al. previously found an increase in hepatic insulin sensitivity following weight reduction in females with lipedema and obesity (Cifarelli et al., 2025). However, the study did not disclose the macronutrient composition of the diet. Moreover, although Jeziorek et al., found no change in HOMA-IR following a 7-month LCD (1681 kcal/day; 7 E% carbohydrate) with a 12 kg weight loss in females with lipedema, the participants exhibited normal baseline HOMA-IR values (Jeziorek et al., 2023), and a higher degree of insulin resistance may have been needed to detect metabolic benefits of the LCD (Lee and Fujioka, 2011). Most studies in individuals with obesity without lipedema show that LCDs produce greater improvements in insulin resistance than low-fat diets (Feng et al., 2025). Volek and colleagues compared a 4-week very-low carbohydrate diet with a 4-week low-fat diet using a balanced crossover design in females with overweight and obesity and found significantly lower fasting glucose and insulin plasma concentrations, as well as lower HOMA-IR after the very-low- carbohydrate diet (Volek et al., 2004).

Both diet groups showed reductions in fasting glucose concentrations, with a significantly greater decrease in the LCD group. This is consistent with previous studies in individuals with overweight and obesity, where LCDs have been associated with improved glycemic control (Feng et al., 2025), and may partly reflect the greater weight loss observed in the LCD group. However, the difference between groups remained significant after adjusting for weight loss, suggesting that the observed difference was not explained by weight loss alone. Nevertheless, residual confounding cannot be completely excluded, and the findings should be interpreted with caution. Future studies should ensure equal weight loss goal in both groups to confirm these observations.

Glucose metabolism in lipedema remains controversial. Females with lipedema appear to have a lower risk of metabolic disease compared to people living with obesity, despite similar BMI (Faerber, 2018; Jeziorek et al., 2025; Nankam et al., 2022). In line with this, elevated HbA1c has been described in females with overweight/obesity, but not in females with lipedema (Mühlberg et al., 2026). Previous intervention studies in lipedema have reported no change in basal glucose concentration despite weight loss (Sørlie et al., 2022; Jeziorek et al., 2023). Sørlie et al. observed no change in basal glucose following 6 weeks of an eucaloric LCD (5–10 E% carbohydrates), despite a mean weight loss of 5 kg (Sørlie et al., 2022). Similarly, Jeziorek et al. observed no change in fasting glucose following 7 months on an LCD (7 E% carbohydrates), despite a 13% weight loss (Jeziorek et al., 2023). Aside from lower baseline insulin sensitivity, these discrepancies may partly be explained by smaller sample sizes in these studies (n = 9 in Sørlie et al. and n = 24 in Jeziorek et al.) compared with the present study (n = 70) (Sørlie et al., 2022; Jeziorek et al., 2023).

Interestingly, however, the LCD group showed a significant increase in postprandial glucose concentrations (iAUC). This response likely reflects the composition of the standardized test meal with a carbohydrate content (72 g) exceeding the participants’ usual daily intake (average of 67 g/day) during the intervention period. This might have resulted in an exaggerated postprandial glycemic response and was accompanied by increases in postprandial C-peptide concentrations, which may be interpreted as a normal physiological response to the glycemic load. Consistent with a normal response, an increase in GIP response in the postprandial state was observed in both groups. This contrasts with a previous study showing reduced postprandial GIP after a 15% weight loss after a very low energy diet in people with obesity (Finn et al., 2025). That study used a shake with a low glycemic index (300 kcal with 26 g carbohydrates) as a test meal, compared to the present study that used real food and a much higher energy and carbohydrate content (600 kcal with 63 g carbohydrates), which may account for at least some of the differences. A study where postmenopausal females with obesity were randomized to either a Paleolithic diet or a healthy control diet based on the Nordic recommendations for 24 months, reported an increase in GIP postprandial concentrations during an oral glucose tolerance test in the Paleolithic group with 10% weight loss, but not in the control group with 6% weight loss (Otten et al., 2019). Again, the energy content and macronutrient composition of the diet may have contributed to the inconsistencies in the findings, along with the differences in weight loss.

We have previously shown in this cohort of patients that an LCD leads to greater reductions in pain perception compared with a low-fat diet (Lundanes et al., 2024a). The superiority of the LCD in reducing pain does not seem to be associated with reductions in inflammation (Lundanes et al., 2025), and we show here that despite greater improvements in insulin dynamics, following the LCD, insulin dynamics does not seem to be involved in the observed reduction in pain. We have previously reported that changes in the plasma concentration of some fatty acids correlate with pain reduction (Lundanes et al., 2026), and given the role of insulin in regulating lipid metabolism it could be expected that also insulin could be involved in pain reduction. However, insulin biology is complex and the measures we used may not have captured specific features of insulin dynamics that may contribute to mechanisms affecting neuron hypersensitivity or other causes of pain in lipedema. Nevertheless, the absence of an observed association does not exclude a contribution from insulin-related pathways. Considering the inconsistencies in the literature regarding insulin and glucose metabolism in females with lipedema, future long-term studies with even more mechanistic assessment of glucose-insulin biology are needed to clarify the metabolic effects of LCDs and their potential role in symptom improvement.

In lipedema, it has been shown that altered microRNA expression may increase sensitivity to the AGEs pathway, which may link glycemic fluctuations to inflammation and symptom severity (Cione et al., 2024). Therefore, dietary interventions that reduce postprandial glycemic response may be especially relevant in lipedema. Even though we did not find group differences regarding inflammation (Lundanes et al., 2025), the effect of the intervention may not depend solely on total carbohydrate intake, but also carbohydrate quality and fiber intake. The fiber intake ranged between 23.1-25.7 g/day in the LCD group and 28.8-30.6 g/day in the low-fat diet group, and was statistically significant lower in the LCD group (Lundanes et al., 2024a). This is supported by a case report in lipedema, that showed that reintroduction of carbohydrates up to 100 g/day in combination with sufficient fiber intake was associated with improvements in pain and quality of life. The authors suggested that this was due to improved glycemic stability (Cannataro et al., 2026).

Physical activity is an important determinant of glycemic and insulin regulation. Although sedentary time increased and light physical activity decreased in both groups during the intervention, no significant between-group differences were observed (Lundanes et al., 2024b). Changes in physical activity may therefore have influenced glycemic and insulin regulation over time but are unlikely to explain the differences observed between the dietary groups.

This study has both strengths and limitations. To our knowledge, this is the first randomized controlled trial investigating insulin dynamics following LCDs vs. low-fat diets in females with lipedema and obesity. The mixed-meal test and postprandial as well as fasting measurements, along with insulin clearance assessed by measuring both insulin and insulin C-peptide, contributes with new insight into glucose-insulin dynamics in lipedema. Another strength is that dietary compliance was high and dropout rate low (Lundanes et al., 2024a). Limitations include the short duration of the dietary intervention, and that the study was powered to investigate changes in pain (Lundanes et al., 2024a) rather than insulin dynamics, and as such might be underpowered to identify differences between diet groups in this outcome measure. Additionally, HOMA-IR and the Matsuda index are only proxies of insulin sensitivity, whereas the gold standard is the euglycemic–hyperinsulinemic clamp (DeFronzo et al., 1979).

## 5 Conclusion

Both LCD and low-fat low-energy diets led to improvements in insulin dynamics, however, the LCD was associated with additional benefits, including a greater reduction in basal glucose concentrations. However, changes in the measured indices of insulin dynamics were not associated with pain reduction, suggesting that the effects of the LCD on pain and systemic metabolic regulation may involve other pathways. Future studies should further explore mechanisms underlying improvements in pain as well as metabolic regulation following LCDs in females with lipedema.

## Data Availability

The raw data supporting the conclusions of this article will be made available by the authors, without undue reservation.
