# Supplementary material for: Glucose and insulin dynamics following low-carbohydrate and low-fat diets in females with lipedema and obesity
Source: Front Cell Dev Biol. 2026 Sep 15;14:1893868. doi: 10.3389/fcell.2026.1893868 (PMC13619572; doi:10.3389/fcell.2026.1893868)
Supplement: Supplementary file 1 [file Table1.docx]

| Supplementary Table 1. Associations between changes in glucose and insulin dynamic variables and group after adjusting for weight loss. | | | |
| --- | --- | --- | --- |
|  | **Coefficient** | **95% CI** | **P value** |
| Glucose |  |  |  |
| Fasting, mmol/L | -0.31 | -0.63 to -0.00 | 0.047 |
| tAUC, mmol/L*min | 51.34 | -17.74 to 120.49 | 0.142 |
| iAUC, mmol/L*min | 101.02 | 37.10 to 164.94 | 0.003 |
| C-peptide |  |  |  |
| Fasting, nmol/L | -0.05 | -0.19 to 0.09 | 0.487 |
| tAUC, nmol/L*min | 29.15 | -36.55 to 94.85 | 0.378 |
| iAUC, nmol/L*min | 38.74 | -18.14 to 95.62 | 0.178 |
| Insulin |  |  |  |
| Fasting, µIU/mL | -0.01 | -6.51 to 4.39 | 0.697 |
| tAUC, µIU/mL*min | 470.02 | -1980.84 to 2920.87 | 0.702 |
| iAUC, µIU/mL*min | 632.70 | -1572.59 to 2837.99 | 0.567 |
| GIP |  |  |  |
| Fasting, pmol/L | -1.55 | -10.81 to 7.72 | 0.739 |
| tAUC, pmol/L*min | 394.53 | -1050.30 to 1839.36 | 0.586 |
| iAUC, pmol/L*min | 649.64 | -1275.72 to 2575.00 | 0.501 |
| Matsuda index |  |  |  |
|  | 0.44 | -0.46 to 1.34 | 0.331 |
| HOMA-IR |  |  |  |
|  | -0.54 | -1.96 to 0.88 | 0.448 |
| Insulin clearance |  |  |  |
|  | 0.43 | -0.89 to 1.75 | 0.518 |
| Data analyzed using multiple regression, with change in glucose and insulin dynamic variables as dependent variable, and weight loss and group as independent variables. Only coefficients from group are presented. CI: Confidence interval. GIP: glucose-dependent insulinotropic polypeptide. HOMA-IR: homeostasis model assessment of insulin resistance. Std.: Standardized. | | | |
